# Prognostic Significance of Baseline Neutrophil Count and Lactate Dehydrogenase Level in Patients With Esophageal Squamous Cell Cancer Treated With Radiotherapy

**DOI:** 10.3389/fonc.2020.00430

**Published:** 2020-04-15

**Authors:** He-San Luo, Hong-Yao Xu, Ze-Sen Du, Xu-Yuan Li, Sheng-Xi Wu, He-Cheng Huang, Lian-Xing Lin

**Affiliations:** ^1^Department of Radiation Oncology, Shantou Central Hospital, Affiliated Shantou Hospital of Sun Yat-sen University, Shantou, China; ^2^Department of Surgical Oncology, Shantou Central Hospital, Affiliated Shantou Hospital of Sun Yat-sen University, Shantou, China; ^3^Department of Medical Oncology, Shantou Central Hospital, Affiliated Shantou Hospital of Sun Yat-sen University, Shantou, China

**Keywords:** prognosis, esophageal squamous cell cancer, definitive radiotherapy, LDH, neutrophil

## Abstract

**Background:** This present study aimed to explore the prognostic value of pretreatment neutrophil and lactate dehydrogenase (LDH) and to develop a prognostic risk scoring model to predict prognosis in esophageal squamous cell cancer (ESCC) patients treated with definitive radiotherapy.

**Methods:** Retrospectively collected data of patients who received definitive radiotherapy for ESCC at Shantou Central Hospital between January 2009 and December 2015 were included for the analysis. The association between the level of LDH and neutrophil and clinicopathological characteristics were analyzed. We performed univariate and multivariate analyses to identify the prognostic predictors for patients with ESCC. Based on the results, we also developed a prognostic risk scoring model and assessed its predictive ability in the subgroups.

**Results:** A total of 567 patients who received definitive radiotherapy for ESCC were included in the present study. The optimal cutoff values were 4.5 × 10^9^/L, 3.25, and 220 U/L for neutrophil, neutrophil-to-lymphocyte ratio (NLR), and LDH, respectively. A high level of LDH was significantly associated with advanced N stage (*p* = 0.031), and neutrophil count was significantly associated with gender (*p* = 0.001), T stage (*p* < 0.001), N stage (*p* = 0.019), clinical stage (*p* < 0.001), and NLR (*p* < 0.001). Multivariate survival analysis identified gender (*p* = 0.006), T stage (*p* < 0.001), N stage (*p* = 0.008), treatment modality (*p* < 0.001), LDH level (*p* = 0.012), and neutrophil count (*p* = 0.038) as independent prognostic factors for overall survival. Furthermore, a new prognostic risk scoring (PRS) model based on six prognostic factors was developed, in which the patients were divided into three groups with distinct prognosis (χ2 = 67.94, *p* < 0.0001).

**Conclusions:** Elevated baseline LDH level and neutrophil count predicted poor prognosis for ESCC patients treated with definitive radiotherapy. A PRS model comprised of LDH, neutrophil count, and other prognostic factors would help identify the patients who would benefit the most from definitive radiotherapy.

## Introduction

Esophageal cancer (EC) is one of the most common digestive malignant tumors, with high recurrence rate and poor overall survival (OS) (1). For patients with early EC, surgery is the mainstay of treatment (2). The majority of patients with locally advanced EC lost the opportunity for surgery at the time of diagnosis. Definitive concurrent chemoradiotherapy (dCRT) has been recommended as a standard treatment and plays important roles in these patients (1). However, the effectiveness of radiotherapy varies greatly among different patients, even patients at the same TNM stage and who received similar radiotherapy regimens, suggesting that there were some other factors affecting the effectiveness of radiotherapy, including patients' characteristics, tumor subsite, and hematological parameters (3–5). To our knowledge, no widely used prediction model about prognosis has been established in patients with esophageal squamous cell carcinoma (ESCC) treated with radiotherapy. Thus, it is critical to identify more accurate prognostic indicators and to develop a reliable prediction model for estimating the prognosis of patients with ESCC treated with radiotherapy.

The inflammation process has been proposed to be an important feature in patients with malignant tumors (6), which is involved in the progression of tumorigenesis, disease development, and patient prognosis (7, 8). Furthermore, some routinely tested blood parameters, such as neutrophil count, lymphocyte count, and lactate dehydrogenase (LDH) level, have been demonstrated as potential inflammatory biomarkers and have prognostic value in patients with cancers (9–11). Neutrophils are acknowledged as the first line of defense against inflammations and infections, as well as play an important role in the tumor microenvironment (TME) (12, 13). Previous studies have shown that tumor-associated neutrophil (TAN) was capable to suppress the immune system in the TME, which results in treatment resistance and promotes cancer development (14, 15). Patients with low neutrophil count were also found to exhibit better radiosensitivity (16). However, the predictive value of neutrophil count in the prognosis of ESCC patients treated with radiotherapy is still unclear. Recently, the prognostic value of LDH has been widely investigated in various cancers, such as metastatic renal cell carcinoma (17), breast cancer (18), nasopharyngeal carcinoma (19), prostate cancer (20), lymphoma (21), non–small cell lung cancer (22), and ESCC (23, 24). Although LDH and neutrophil count are reliable prognostic predictors, it is still not clear whether they can be combined together in a prognostic risk score model to predict the prognosis of ESCC patients treated with radiotherapy.

In this study, we aimed to explore the role of neutrophil count and LDH level in the prognosis of patients with ESCC treated with radiotherapy. We performed univariate and multivariate analyses to identify the prognostic factors for the ESCC patients. According to the results of the multivariate analysis, we devised a prognostic risk scoring model for estimating the prognosis of ESCC patients treated with radiotherapy.

## Patients and Methods

### Study Design

We retrospectively reviewed the patients receiving definitive radiotherapy for EC at the Department of Radiation Oncology, Shantou Central Hospital during the period from January 2009 to December 2015. Only patients pathologically diagnosed as ESCC were included in this study. Patients with non-ESCC tumors were excluded from this study. The remaining patients were excluded if they met the following exclusion criteria: (1) patients with distant metastatic disease; (2) patients who received low-dose palliative radiotherapy (< 50.4 Gy for patients treated with radiotherapy without chemotherapy and < 60 Gy for patients treated with chemoradiotherapy); (3) patients who received preoperative or postoperative adjuvant radiotherapy; (4) patients who had recurrent disease and received radiotherapy for salvage purposes; (5) patients who failed to complete therapy; and (6) patients who had other primary tumor. This study was approved by the Institutional Committee of the Shantou Central Hospital on Human Rights. Disease of the patients was staged according to the sixth edition of AJCC TNM classification for EC.

### Radiotherapy Protocols

Radiotherapy was delivered by three-dimensional conformal radiation therapy or intensity-modulated radiation therapy technique in this study. A Varian IX or Varian 23EX linear accelerator was used to deliver the radiotherapy treatment plan. The treatment planning approach has been reported in our previous study (25). Briefly, the gross tumor volume (GTV) includes the EC (GTVp) and the positive regional lymph nodes (GTVnd). The delineation of GTV was determined by CT, barium esophagogram, endoscopic examination, or PET imaging. The GTVp plus a 0.5–1cm radial margin and a 2.5–3 cm proximal and distal margin and the GTVnd plus a 0.5–0.8 cm margin were defined as CTV. The planning target volume (PTV) encompassed the CTV plus a 0.5–1 cm margin. All patients received simultaneous integrated boost (SIB) radiotherapy, which had been reported in a recent phase 1/2 trial conducted by Chen et al. (26). The prescribed dose was 60–66 Gy to GTV in 28–30 fractions, five fractions per week, and at least 50.4 Gy to CTV in 28 fractions, five fractions per week. Two cycles of platinum-based chemotherapy combined with 5-fluorouracil or a taxane (docetaxel or paclitaxel) were administered on the patients concurrently with radiotherapy.

### Data of Hematological Index Collection

The pretreatment data of neutrophil count, lymphocyte count, and LDH level were collected from the test reports. The cutoff value for the LDH level was the upper limit of normal (ULN) values set (220 U/L) of the biochemical detector used in our hospital. The neutrophil count divided by the lymphocyte count was defined as the neutrophil-to-lymphocyte ratio (NLR).

### Follow-Up

All patients were assessed weekly during radiotherapy to monitor the treatment toxicities. Physical examination, blood routine, and biochemical test were done at a weekly visit. The first follow-up was 1 month after finishing radiotherapy, then continuing every 3 months for 2 years and every 6–12 months until disease progression or death. The last follow-up date was May 31, 2019. Physical examination, blood routine and biochemical test, barium esophagogram, and contrast-enhanced CT scan of the neck, chest, and abdomen were done at each follow-up visit. Information on patients' clinicopathological characteristics was retrospectively collected from their medical records.

### Statistical Analysis

Recurrence-free survival (RFS) was defined as the interval from the date of definitive radiotherapy to either the first evidence of any recurrence (local or distant metastases) or death. OS was calculated from the date of treatment beginning to either the date of death from any cause or last follow-up. A chi-square test was performed to compare the differences of patients' clinicopathological characteristics. RFS and OS rates were estimated using the Kaplan–Meier method, and survival curve comparisons were performed using the log-rank test. Multivariate analysis was performed using a Cox regression model to identify prognostic factors associated with OS. The optimal cutoff value for NLR and neutrophil count to distinguish the difference of complete response (CR) rate was determined using the receiver operating characteristics (ROC) curve analysis. A two-sided *P* < 0.05 was considered statistically significant. All statistical analysis and data management were done with the statistical software IBM SPSS v22.0 (SPSS Inc., Chicago, IL, USA).

## Results

### Patient Characteristics

A total of 567 ESCC patients who received definitive radiotherapy for ESCC in our hospital were included in this study, with 413 (72.8%) men and 154 (27.2%) women. The patient characteristics including age, gender, tumor location, T stage, N stage, TNM stage, and treatment modality are summarized in Table 1. All the patients received definitive radiotherapy, with a radiation dose ranging from 50 to 78 Gy. Two hundred and forty-seven (43.6%) patients received definitive radiotherapy alone, and 320 (56.4%) patients received definitive concurrent chemoradiotherapy. There were 209 (36.9%) patients who achieved CR after radiotherapy.

**Table 1:** Baseline patient characteristics.

**Characteristics**	**Number (*n* = 567)**
Age (years), median	64 (40–95)
≦65y	298
>65y	269
**Gender**	
Female	154
Male	413
**Location**	
Cervical	37
Upper thoracic	125
Middle thoracic	336
Lower thoracic	69
**T stage**	
T1	9
T2	152
T3	146
T4	260
**N stage**	
N0	119
N1	448
**TNM stage**	
I+II	238
III+IV	329
**Treatment**	
RT	247
CCRT	320
RT dose (Gy), median	64 (50–78)
≦64 Gy	313
>64 Gy	254
**Complete response**	
Yes	209
No	358
NLR, median	2.64 (0.60–31.67)
LDH (U/L), median	208 (83.0–617.0)
Neutrophils (10^9^/L), median	4.8 (1.1–15.8)

### Baseline Serum LDH Level, Neutrophil Count, and Clinicopathological Characteristics

At baseline, the pretreatment blood routine and blood biochemical examination were performed in all 567 patients. The median LDH was 208.0 U/L, ranging from 83.0 to 617.0 U/L. The default normal range of LDH was 80–220 U/L according to the biochemical detector used in our hospital. The neutrophil count ranged from 1.1 to 15.8 × 10^9^/L, with a median of 4.8 × 10^9^/L. The pretreatment NLR was calculated by the formula of the neutrophil count divided by the lymphocyte count. The median pretreatment NLR was 2.64, ranging from 0.60 to 31.67. The ROC curve was used to determine the NLR and neutrophil count thresholds to predict CR. The optimal cutoff values to predict CR were 4.5 × 10^9^/L and 3.25 for neutrophil count and NLR, respectively. The LDH threshold was determined to be 220 U/L according to the upper limit of normal. Using these cutoff values, we stratified the patients into different groups (LDH ≦ 220 U/L vs. LDH>220 U/L and neutrophil ≦ 4.5 × 10^9^/L vs. neutrophil >4.5 × 10^9^/L, respectively; as shown in Table 2). As a result, 347 patients had a low level of LDH (≦ 220 U/L), and 220 patients had a high level of LDH (>220 U/L). Two hundred and fifty-one patients had a low count of neutrophil (≦ 4.5 × 10^9^/L), and 316 patients had a high count of neutrophil (>4.5 × 10^9^/L). A high level of LDH was significantly associated with the advanced N stage (*p* = 0.031), and neutrophil count was significantly associated with gender (*p* = 0.001), T stage (*p* < 0.001), N stage (*p* = 0.019), clinical stage (*p* < 0.001), and NLR (*p* < 0.001).

**Table 2:** The association between levels of LDH and neutrophil and clinicopathological characteristics in patients with ESCC.

**Characteristics**		**LDH (U/L)**				**Neutrophil (10**^****9****^**/L)**			
	**≦220**	**>220**	**χ2 /t**	***p***	**≦4.5**	**>4.5**	**χ2 /t**	***p***
Age (years)			0.637	0.425			2.2812	0.131
≦65y	187 (53.9)	111 (50.5)			123 (49)	175 (55.4)		
>65y	160 (46.1)	109 (49.5)			128 (51)	141 (44.6)		
Gender			0.190	0.663			10.231	0.001
Female	92 (26.5)	62 (28.2)			85 (33.9)	69 (21.8)		
Male	255 (73.5)	158 (71.8)			166 (66.1)	247 (78.2)		
Location			1.919	0.589			2.172	0.538
Cervical	19 (5.5)	18 (8.2)			13 (5.2)	24 (7.6)		
Upper thoracic	79 (22.8)	46 (20.9)			60 (23.9)	65 (20.6)		
Middle thoracic	205 (59.1)	131 (59.5)			146 (58.2)	190 (59.3)		
Lower thoracic	44 (12.7)	25 (11.4)			32 (12.7)	37 (12.2)		
T stage			2.166	0.539			35.330	< 0.001
T1	6 (1.7)	3 (1.4)			5 (2.0)	4 (1.3)		
T2	95 (27.4)	57 (25.9)			90 (35.9)	62 (19.6)		
T3	82 (23.6)	64 (29.1)			75 (29.9)	71 (22.5)		
T4	164 (47.3)	96 (43.6)			81 (32.3)	179 (56.6)		
N stage			4.635	0.031			5.525	0.019
N0	83 (23.9)	36 (16.4)			64 (25.8)	55 (28.7)		
N1	264 (76.1)	184 (83.6)			187 (74.2)	261 (71.3)		
TNM stage			0.004	0.952			17.822	< 0.001
I+II	146 (42.1)	92 (19.4)			130 (51.8)	108 (34.2)		
III+IV	201 (57.9)	128 (45.2)			121 (48.2)	208 (65.8)		
NLR			0.920	0.337		59.839	< 0.001	
≦3.25	239 (68.9)	143 (65.0)		212 (84.5)	170 (53.8)			
>3.25	108 (31.1)	77 (35.0)		39 (15.5)	146 (46.2)			

### The Association Between LDH Level, Neutrophil Count, and Treatment Outcome

**Figure 1:**
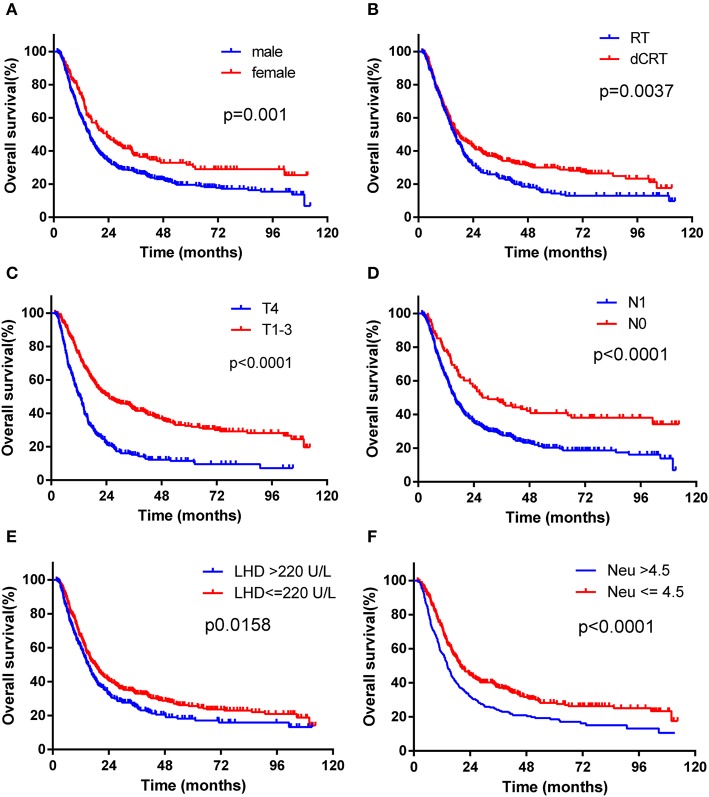
Kaplan-Maier survival curves of overall survival of ESCC patients treated with radiotherapy stratified according to different prognostic factors. **(A)** Patients were stratified by gender. **(B)** Patients were stratified by treatment modality (RT vs. CCRT). **(C**) Patients were stratified by T stage (T4 stage vs. T1-3 stage). **(D)** Patients were stratified by N stage (N1 vs. N0 stage). **(E)** Patients were stratified by LDH level (LDH > 220 U/L vs. LDH _220 U/L). **(F)** Patients were stratified by neutrophil count (neutrophil >4.5G/L vs neutrophil ≤ 4.5G/L).

Median follow-up was 67.4 months (95% CI, 56.6–73.4 months) in this study cohort. The median OS was 16.4 months (95% CI, 15.3–18.5 months). We performed univariate and multivariate analyses to identify the prognostic factors. Univariate analysis showed that gender (*p* = 0.001), tumor location (*p* = 0.001), T stage (*p* < 0.001), N stage (*p* < 0.001), treatment modality (*p* = 0.002), LDH level (*p* = 0.010), neutrophil count (*p* < 0.001), and NLR (*p* = 0.001) were associated with RFS. In the following multivariate analysis, gender (*p* = 0.004), T stage (*p* < 0.001), N stage (*p* = 0.005), treatment modality (*p* < 0.001), LDH level (*p* = 0.007), and neutrophil count (*p* = 0.037) were found to be independently associated with RFS (Table 3). Furthermore, in the univariate analysis, gender (*p* = 0.001), tumor location (*p* < 0.001), T stage (*p* < 0.001), N stage (*p* < 0.001), treatment modality (*p* = 0.004), LDH level (*p* = 0.016), neutrophil count (*p* < 0.001), and NLR (*p* < 0.001) were associated with overall survival. In the multivariate analysis, gender (*p* = 0.006), T stage (*p* < 0.001), N stage (*p* = 0.008), treatment modality (*p* < 0.001), LDH level (*p* = 0.012), and neutrophil count (*p* = 0.038) were still independently associated with overall survival (Table 4). The prognostic impacts on overall survival of gender (*p* = 0.001), treatment modality (*p* = 0.0037), T stage (*p* < 0.0001), N stage (*p* = 0.0001), LDH level (*p* = 0.0158), and neutrophil count (*p* < 0.0001) are shown in Figures 1A–F, respectively.

**Table 3:** Univariate and multivariate analysis of clinical factors associated with Recurrence-Free Survival among patients with ESCC.

**Variates**	**Univariate analysis**			**Multivariate analysis**		
	**HR (95%CI)**	**χ2**	***p***	**HR (95%CI)**	**χ2**	***p***
Gender	0.694(0.559–0.862)	10.870	0.001	0.717 (0.573–0.898)	8.444	0.004
Age	1.032 (0.857–1.243)	0.112	0.738			
Location	1.258 (1.104–1.433)	11.925	0.001		3.536	0.316
Cervical				Reference		
Upper thoracic				1.171 (0.750–1.830)	0.482	0.488
Middle thoracic				1.331 (0.880–2.013)	1.832	0.176
Lower thoracic				1.474 (0.919–2.364)	2.597	0.107
T stage	1.479 (1.320–1.657)	45.647	0.000		27.225	0.000
T4				Reference		
T1				0.386 (0.166–0.897)	4.890	0.027
T2				0.595 (0.462–0.765)	16.269	0.000
T3				0.563 (0.437–0.724)	20.028	0.000
N stage	1.799 (1.408–2.299)	22.011	0.000	1.449 (1.116–1.881)	7.755	0.005
RT dose	1.063 (0.883–1.280)	0.414	0.520			
Treatment	0.749 (0.622–0.901)	9.346	0.002	0.628 (0.518–0.762)	22.314	0.000
LDH	1.280 (1.060–1.546)	6.589	0.010	1.304 (1.076–1.580)	7.317	0.007
Neutrophils	1.427 (1.182–1.723)	13.668	0.000	1.242 (1.013–1.522)	4.352	0.037
NLR	1.389 (1.142–1.688)	10.873	0.001	1.021 (0.825–1.264)	0.037	0.848

**Table 4:** Univariate and multivariate analysis of clinical factors associated with Overall Survival among patients with ESCC.

**Variates**	**Univariate analysis**			**Multivariate analysis**		
	**HR (95%CI)**	**χ2**	***p***	**HR (95%CI)**	**χ2**	***p***
Gender	0.695 (0.558–0.866)	10.536	0.001	0.727 (0.580–0.911)	7.648	0.006
Age	1.048 (0.868–1.265)	0.239	0.625			
LocationCervical	1.274 (1.116–1.454)	12.887	0.000	Reference	3.534	0.316
Upper thoracic				1.154 (0.733–1.819)	0.383	0.536
Middle thoracic				1.319 (0.865–2.011)	1.650	0.199
Lower thoracic				1.471 (0.910–2.379)	2.481	0.115
T stage	1.525 (1.358–1.713)	50.716	0.000		32.151	0.000
T4				Reference		
T1				0.367 (0.158–0.852)	5.443	0.020
T2				0.568 (0.439–0.735)	18.490	0.000
T3				0.527 (0.408–0.680)	24.268	0.000
N stage	1.799 (1.389–2.280)	20.741	0.000	1.430 (1.099–1.861)	7.077	0.008
RT dose	1.052 (0.871–1.270)	0.274	0.601			
Treatment	0.758 (0.628–0.914)	8.360	0.004	0.638 (0.524–0.776)	20.177	0.000
LDH	1.265 (1.044–1.531)	5.772	0.016	1.283 (1.076–1.580)	6.278	0.012
Neutrophils	1.462 (1.208–1.771)	15.158	0.000	1.245 (1.012–1.532)	4.308	0.038
NLR	1.426 (1.171–1.738)	12.439	0.000	1.014 (0.8175–1.259)	0.016	0.899

### A New Prognostic Risk Scoring Model Based on LDH Level and Neutrophil Count

**Figure 2:**
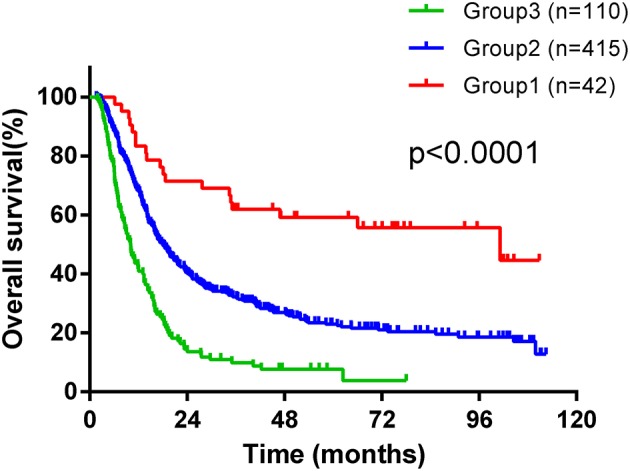
Kaplan-Maier survival curves of overall survival of ESCC patients treated with radiotherapy stratified according to a new prognostic risk scoring (PRS) model.

We devised a new prognostic risk scoring (PRS) model based on gender, treatment modality, T stage, N stage, LDH level, and neutrophil count, which were identified as independent prognostic factors in multivariate analysis for OS. In the PRS model, patients with none or one to two of these poor prognostic factors were scored as one (Group one), patients with three or four of these poor prognostic factors were scored as two (Group two), and patients with five or six of these poor prognostic factors were scored as three (Group three). According to this PRS model, patients were stratified into three groups with distinct prognosis, with 42 (7.4%) patients in Group one, 415 (73.2%) patients in Group two, and 110 (19.4%) patients in Group three. The median OS time was 101.2 months in Group one, which was significantly longer than 18 months in Group two and 10.05 months in Group three (shown in Figure 2, χ^2^ = 67.94, *p* < 0.0001). Moreover, the CR rate in Group one was significantly higher than that in Group two and Group three (χ^2^ = 24.031, *p* < 0.0001). Twenty-three (54.8%) patients achieved CR in Group one, 166 (40%) patients achieved CR in Group two, and 20 (18.2%) patients achieved CR in Group three.

## Discussion

For patients with ESCC treated with surgery, TNM stage classification acts as the most important prognostic factor for many years. However, TNM stage classification seemed not sufficient to present enough prognostic information for patients treated with definitive radiotherapy (27). There could be some other factors impacted on the prognosis of patients who received definitive radiotherapy. Thus, identification of other new prognostic factors could allow a better prediction for treatment outcome. To further explore prognostic factors to identify patients with different prognosis, more easily available prognostic factors are warranted.

Neutrophil count and LDH both routinely detected the hematological index and were easily available in our clinical practice. Previous studies have investigated the prognostic value of LDH level, neutrophil count, and NLR in many solid tumors (10, 21, 24). However, there was no investigation about the role of the LDH level combined with neutrophil count or NLR in the prognosis of ESCC patients treated with radiotherapy. This study aimed to investigate the prognostic value of the LDH level, neutrophil count, and NLR in ESCC patients treated with radiotherapy. What is more, for the first time, we established a new risk prognostic scoring model based on the baseline LDH level and neutrophil count, which stratified patients into three groups with different prognosis.

According to previous studies, systemic inflammation was an enabling characteristic for cancer development and promoted tumor progression by affecting the response to systemic therapies (6, 28). NLR, determined by the neutrophil count and lymphocyte count, was suggested to reflect the systemic inflammatory responses (29). Previous study has reported that NLR could serve as a prognostic indicator for survival in EC (30). An investigation that enrolled a relatively large population of ESCC patients from Chen et al. (31) revealed that pretreatment elevated NLR was significantly associated with an advanced clinical stage and reduced OS. Moreover, elevated NLR was an independent prognostic indicator for OS in patients receiving chemoradiotherapy but not those receiving surgery. Interestingly, in this present study, multivariate analysis showed that NLR was not an independent prognostic indicator for RFS and OS in ESCC patients treated with radiotherapy. However, increased neutrophil was significantly associated with advanced T stage, N stage, clinical stage, and poor OS in ESCC patients treated with radiotherapy. One possible explanation is that tumor microenvironment is influenced by neutrophils themselves, but NLR is affected by lymphocyte count and couldn't reflect changes in the tumor microenvironment. Another possibility is that NLR and neutrophil count interact with each other in the modeling stats. When combined with other prognostic factors in multivariate analysis, neutrophil count had stronger predictive ability compared with NLR. Based on the results, pretreatment neutrophil count might be more appropriate to be used as a prognostic factor than NLR and could be a useful baseline indicator to predict the outcome for ESCC patients treated with radiotherapy.

Growing evidence has showed that neutrophilia can occur in cancer patients. Moreover, neutrophils are thought to promote angiogenesis and tumor growth, degrade the extracellular matrix, provide favorable conditions for metastasis, and potentiate genome instability and tumor evolution (29). Neutrophils can also be localized to the tumor to establish tumor-associated neutrophil (TAN), resulting in treatment resistance and cancer development (15). In this study, we explored the optimal cutoff value of neutrophil count using the ROC curve analysis to predict CR in ESCC patients treated with radiotherapy and found that patients with high neutrophil count had poor RFS and OS, indicating that increased neutrophil count may be a predictor for poor radiosensitivity.

According to previous studies, an elevated level of LDH isoforms is more common in malignant tumors than normal cells (32). The increased LDH level could promote tumor progression by regulating the tumor metabolism and microenvironment and acts as a poor prognostic indicator for cancer patients (32, 33). A meta-analysis investigating the prognostic value of the LDH level in solid tumors showed that a high LDH level is associated with poor survival in melanoma, gastric, lung cancer, prostate, and renal cell carcinomas (34). Recently, a high LDH level has been demonstrated to effectively predict the response to cancer treatment, such as chemotherapy (11), anti-angiogenetic agents (35), and checkpoint immunotherapy (22, 24) in various cancers. The prognostic role of the LDH level was also investigated in ESCC patients who underwent curative treatment in the study from Wei et al. (23). However, the study included patients treated with surgery or chemoradiotherapy, which led to treatment bias. In this present study, we only included the ESCC patients treated with radiotherapy and demonstrated that an elevated LDH level was an indicator for poor prognosis in the setting.

Several limitations were inevitable in our study. First, the retrospective nature of this study led to selection bias and potential confounding biases. Second, there were some other prognostic factors influencing the level of LDH and neutrophil count such as infectious diseases, which could not be stratified in our retrospective study, and thus the implication of the LDH level and neutrophil count on the prognosis of ESCC patients treated with radiotherapy should be further investigated in a carefully designed study. Third, some patients who cannot tolerate concurrent chemoradiotherapy were treated with radiotherapy only, leading to treatment selection bias. Thus, we performed multivariate analysis to identify independent prognostic factors. Finally, the conclusions were based on only a small number of 567 patients treated with radiotherapy. It is inappropriate to extrapolate to the patients in a trimodality setting.

In conclusion, we provided an investigation about the prognostic significance of the LDH level and neutrophil count in ESCC patients treated with radiotherapy and the optimal cutoff value to predict the response to radiotherapy. Furthermore, we demonstrated that a high level of LDH and neutrophil count were associated with poor prognosis in ESCC patients, and proposed a prognostic risk scoring model based on the LDH level and neutrophil count to help estimate the prognosis for ESCC patients for the first time.

## Data Availability Statement

The datasets used and/or analyzed during the current study are available from the corresponding author upon reasonable request.

## Ethics Statement

The studies involving human participants were reviewed and approved by Ethics Committee of the Shantou Central Hospital. Written informed consent for participation was not required for this study in accordance with the national legislation and the institutional requirements.

## Author Contributions

H-CH and L-XL designed the study. H-SL prepared figures and wrote the manuscript text. H-YX and S-XW collected the follow-up data. X-YL and Z-SD made statistical analysis. All authors reviewed the manuscript and approved the final manuscript.

### Conflict of Interest

The authors declare that the research was conducted in the absence of any commercial or financial relationships that could be construed as a potential conflict of interest.

## Abbreviations

LDH: lactate dehydrogenase
ESCC: esophageal squamous cell cancer
dCRT: definitive chemoradiotherapy
PRS: prognostic risk scoring
EC: esophageal cancer
OS: overall survival
EAC: esophageal adenocarcinoma
GTV: gross tumor volume
GTVnd: nodal gross tumor volume
CTV: clinical target volume
CTVt: tumor clinical target volume
CTVnd: nodal clinical target volume
PTV: planning target volume
NLR: neutrophil-to-lymphocyte ratio
ROC: receiver operating characteristics
RFS: recurrence-free survival
CR: complete response.
